# Transcriptomic Adjustments of *Staphylococcus aureus* COL (MRSA) Forming Biofilms Under Acidic and Alkaline Conditions

**DOI:** 10.3389/fmicb.2019.02393

**Published:** 2019-10-18

**Authors:** Georgios Efthimiou, George Tsiamis, Milton A. Typas, Katherine M. Pappas

**Affiliations:** ^1^Department of Genetics and Biotechnology, Faculty of Biology, National and Kapodistrian University of Athens, Athens, Greece; ^2^Department of Environmental Engineering, University of Patras, Agrinio, Greece

**Keywords:** MRSA, biofilm, alkaline, acidic, microarray, transcription factors

## Abstract

Methicillin-resistant *Staphylococcus aureus* (MRSA) strains are important human pathogens and a significant health hazard for hospitals and the food industry. They are resistant to β-lactam antibiotics including methicillin and extremely difficult to treat. In this study, we show that the *Staphylococcus aureus* COL (MRSA) strain, with a known complete genome, can easily survive and grow under acidic and alkaline conditions (pH5 and pH9, respectively), both planktonically and as a biofilm. A microarray-based analysis of both planktonic and biofilm cells was performed under acidic and alkaline conditions showing that several genes are up- or down-regulated under different environmental conditions and growth modes. These genes were coding for transcription regulators, ion transporters, cell wall biosynthetic enzymes, autolytic enzymes, adhesion proteins and antibiotic resistance factors, most of which are associated with biofilm formation. These results will facilitate a better understanding of the physiological adjustments occurring in biofilm-associated *S. aureus* COL cells growing in acidic or alkaline environments, which will enable the development of new efficient treatment or disinfection strategies.

## Introduction

*Staphylococcus aureus* is a Gram-positive cluster-forming aerobic coccus that is commonly found on the skin and the respiratory tract of humans and animals. It is recognized as a cause of serious nosocomial infection and especially methicillin-resistant *S. aureus* (MRSA) strains are considered a major public health hazard. MRSA is prevalent in hospitals, prisons, and nursing homes, where people with open wounds, invasive devices such as catheters, and weakened immune systems are at greater risk of nosocomial infection. *S. aureus* is known to efficiently colonize the biomaterials that are used for medical implants and devices. In the event of a biomaterial-associated infection, the device must be substituted, something that seriously burdens the patient, while relapsing infections remain possible. In cases where the device cannot be substituted, the patient faces a higher mortality risk (Arciola et al., 2012). In addition, MRSA strains have been found present in retailed meat products (O’Brien et al., 2012), dairy products (Normanno et al., 2007), seafood (Kumar et al., 2016), green leafs of pre-cut salads (Doulgeraki et al., 2017), the hands of food industrial workers (Kamal et al., 2013) and the equipment and surfaces related to food preparation (Gibson et al., 1999), therefore explaining the alarmingly increasing reports on food-borne acquired MRSA outbreaks (Jones et al., 2002; Harris et al., 2010; Centers for Disease Control and Prevention, 2018). Only in the United States, 72,444 cases of MRSA infections were reported in 2014, while the morbidity rate reached 11.8% (Hassoun et al., 2017).

*Staphylococcus aureus* strains have the ability to form biofilms (BFs), multicellular communities covered by a thick polysaccharide layer, which contribute significantly to antibiotic and detergent resistance (Christensen et al., 1994; Götz, 2002; de Souza et al., 2014). In general, bacterial BFs are multi-layered complex communities which in their mature form contain specific three-dimensional structures that are separated by fluid channels. Depending on the position of cells, they are allowed to differentially express proteins throughout the BF. The formation of BFs is generally regarded as a four-step process that includes: (a) an initial attachment of cells to the surface through ionic or hydrophobic interactions, (b) the accumulation in multiple bacterial layers, mediated by microbial surface components recognizing adhesive matrix molecules (MSCRAMMs), (c) BF maturation with the production of extracellular capsular exopolysaccharide (PNAG) and several exoproteins which mediate the attachment of *S. aureus* cells on surfaces and eDNA, rendering encapsulated cells resistance to phagocytosis and antibiotics, and (d) detachment of BF cells and dispersal in a planktonic state form to initiate a new cycle of BF formation elsewhere, guided by numerous environmental signals, signal transduction pathways and effectors (Patti et al., 1994; Stoodley et al., 2002; Bischoff et al., 2004; Archer et al., 2011; Arciola et al., 2012; Atwood et al., 2015).

The effect of environmental pH on BF formation can influence several important biological processes. For example, wound pH is known to gradually decrease while the wound is healing, due to lactic acid production and other factors. Bacterial BFs can lead to serious infection, if they are tolerant to low pH or antiseptics (Percival et al., 2014; Jones et al., 2015). Moreover, acidic and alkaline detergents are frequently used to decontaminate clinical surfaces and surgical instruments (Lemmer et al., 2004), as well as food-processing surfaces and equipment (Sharma and Beuchat, 2004; Akbas and Cag, 2016). Acidic or alkaline sanitizers are also used to disinfect fruit and vegetables (Park et al., 2011) and orthopedic hardware (Moussa et al., 1996), conditions that can easily allow the survival of tolerant BF-forming bacteria and cause infections.

*Staphylococcus aureus* BF cells exhibit a different phenotype with respect to bacterial physiology, metabolism and gene transcription compared to planktonic cells (Donlan and Costerton, 2002). The ability of *S. aureus* to form BF and its morphology were strongly influenced by significant pH changes (Jones et al., 2015). When weakly acidic and alkaline detergents were used against *S. aureus* BFs on stainless steel surfaces, BF-associated cell numbers were reduced, but the BFs were not completely removed (Ueda and Kuwabara, 2007). Lastly, alkaline and acidic pHs were shown to inhibit *S. aureus* BF formation and reduced its amount and thickness (Nostro et al., 2012).

A number of transcriptomic studies using planktonic *S. aureus* cells that grew in liquid media with acidic or alkaline pH have been published (Weinrick et al., 2004; Bore et al., 2007; Anderson et al., 2010; Rode et al., 2010). These reports have identified few genes whose expression is affected by pH changes, but have not clearly defined specific functional or regulatory mechanisms yet, neither have they contributed to the transcriptomic adjustments occurring in BF-associated MRSA cells growing under acidic and alkaline conditions. Thus, the aim of this study was to detect genes that are differentially expressed in *S. aureus* COL BF cells, under acidic and alkaline conditions (pH5 and pH9, respectively) with the use of DNA microarrays. Two modes of growth were studied: BF-associated cells on a porous nitrocellulose membrane placed on solid media and planktonic cells in liquid medium. Gene expression levels at environments of different pH and growth modes were measured and compared, in order to gain better knowledge about the molecular mechanisms connecting pH-related stress response with BF-formation and pathogenicity in this important human pathogen. This study will help understanding how the pathogen survives and responds under acidic and alkaline conditions, which will lead to the design of better treatment or disinfection strategies.

## Materials and Methods

### Bacterial Strains, Media, and Cultures

Tryptone Soya Broth (TSB) and Tryptone Soya Agar (TSA) were used for growing *S. aureus* COL (MRSA) in this study. 100 μL of an overnight pre-culture were used to inoculate 10 mL of the same medium in sterile glass shake flasks. The flasks were incubated for 5 h at 37°C (150 rpm). HCl and NaOH solutions (1 M) were used to adjust the pH. Colony forming units per mL of liquid culture were determined by serial dilutions and colony enumeration (at least three biological replicates in each case). Biomass from these cultures was harvested for RNA isolation, immediately dissolved in RNAlater^®^ reagent (Ambion, United States), as advised by the manufacturer, and stored at −80°C for further use.

### Biofilm Formation

Four 100 μL drops of a 5-h pre-culture were pipetted on a nitrocellulose membrane (pore size 0.45 μm; Sartorius, United Kingdom), which was placed on TSA with different pH (5, 7, and 9) and allowed to grow statically for 24 h at 37°C. Determination of colony forming units per nitrocellulose disk (at least three biological replicates in each case) and biomass harvestation with RNAlater^®^ reagent were performed as described above.

### Total RNA Extraction and First-Strand cDNA Synthesis

Biomass pellets treated with RNAlater^®^ reagent were dissolved in an aqueous solution of lysostaphin (0.2 U/μL) and incubated at 37°C for 30 min. The samples were transferred into new Eppendorf tubes containing 0.2 g of glass beads (0.6 mm diameter), 750 μL of RA1 lysis buffer (Nucleospin^®^ RNA II kit; Cat. No. 740955.50; Macherey-Nagel, Germany) and 1% β-mercaptoethanol (Sigma-Aldrich, United Kingdom). The samples were vortexed thrice for 30 sec and total RNA was then isolated as suggested by the Nucleospin^®^ RNA II kit instructions. Two elution steps were performed at the end of the procedure. The quality of the extracted RNA was determined by spectrophotometry and gel electrophoresis in a 1.4% agarose gel with DEPC-treated distilled water. First-strand cDNA synthesis was performed by using the PrimeScript^TM^ 1st strand cDNA Synthesis Kit (Cat. No. 6110A; Takara, Japan).

### DNA Microarrays

1.5 μg of synthesized first-strand cDNA were hybridized on a GeneChip^®^ *S. aureus* Genome Array (Cat. No. 900514; Affymetrix, United States), following the procedure suggested by the GeneChip^®^ Expression Analysis Technical Manual (Affymetrix; P/N 702232 Rev. 3). Biological duplicates were used (*n* = 2).

### Microarray Data Analysis

The raw microarray data were first normalized by using the statistical language R (TM4 protocol at: https://github.com/dfci-cccb/www.tm4.org/blob/master/normalizing.html). Data filtering was performed by using MeV (MultiExperiment Viewer Quickstart Guide v. 4.2). The selected variance filter value was 50. Finally, the filtered data were exported to Excel and the Log_2_ ratios of the average gene expression values of the two compared conditions were calculated [Log_2_(Expr1/Expr2)]. A two-tailed paired *t*-test was also performed in Excel, using the gene expression values of each gene for the two compared conditions. Gene expression differences were considered to be significant only if the *p*-value was <0.05. Databases KEGG and Aureowiki were used for confirming gene annotation and function. For gene annotation, the files provided by Affymetrix for this specific microarray product were used^1^.

## Results

*Staphylococcus aureus* can either grow planktonically in the bloodstream or colonize body surfaces, such as the nasopharyngeal mucosa. During the second mode of growth, the pathogen is using cell surface proteins to attach on specific mucosal ligands and then produces a polysaccharide layer that stabilizes and protects the bacterial colony. It was shown by preliminary results that *S. aureus* COL can grow well and form BF even at extreme pH environments (pH 4-10; Efthimiou et al., 2015). Therefore, we chose to study gene expression at pH5 and pH9, as the BF levels on polystyrene surfaces were comparable, although slightly lower, with these at pH7.

**FIGURE 1:**
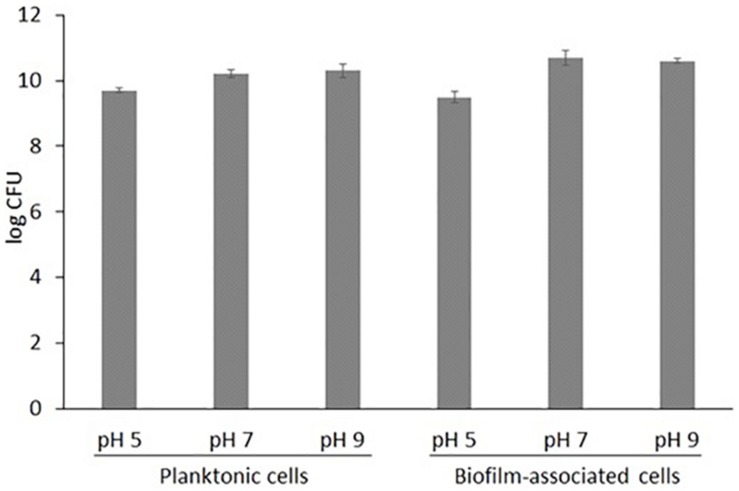
Total growth of *S. aureus* COL in liquid TSB medium (log CFU in 10 mL) and nitrocellulose filters placed on solid TSA medium (log CFU per disk) (*n* = 3, where *n* the number of biological replicates; ± STDEV error bars are also shown).

In this study, *S. aureus* COL (genome sequence PRJNA238) grew well in both liquid and solid media, under acidic, neutral and alkaline conditions. Total planktonic growth reached 10^9^ in acidic and 10^10^ CFU/10 mL in neutral and alkaline TSB and TSA, respectively (*p*-value = 0.0009) (Figure 1). The same was observed for biofilm growth. This indicates that the pathogen is possibly more tolerant to highly alkaline than acidic environments.

Out of the over 3,300 open reading frame genes imprinted on the array (Cat No. 900154) eight were found to be over-expressed in BF-forming *S. aureus* COL cells growing under alkaline conditions. They all had a log_2_ fold-change ratio above 3.16 and a *p*-value < 0.0061 (Table 1A). These genes encoded three transcriptional regulators (CodY, MecA and CtsR), one capsular biosynthesis enzyme (CapC) and four other proteins (two cell wall proteins IsdC or SirD and IsaB; the von Willebrand matrix secreted surface protein, VWbp; and a hypothetical protein similar to SceD, which is involved in autolysis). Under acidic conditions, eleven genes were over-expressed in BF*S. aureus* COL cells and all of them had a log_2_ fold-change ratio above 3.12 and a *p*-value < 0.0316 (Table 2A). These encoded three genes coding for cell surface proteins (MapW, Efb/FnbA and the secreted VWbp), two cell wall-associated proteins (CapC and EbsB), an enzyme with possible phosphomannomutase activity (PgcA) and an ABC transporter (CymR). Additionally, genes coding for a terminase (SACOL 0366), a methionine import protein (MetN2), a hypothetical protein (SACOL2556) and a probable type I site-specific deoxyribonuclease LldI chain (HsdM) were up-regulated.

**TABLE 1:** Significantly upregulated genes at pH 9 in *S. aureus* COL biofilm-associated **(A)** and planktonic cells **(B)** (*p* < 0.05).

**Function**	**Gene name**	**Gene number (in *S. aureus* COL)**	**Gene annotation**	**Log_2_ fold change**	***p*-Value**
**(A)**					
Transcription regulators	*codY*	SACOL1272	pir| BS9899 transcription pleiotropic repressor CodY	3.35	0.0043
	*mecA*	SACOL1003	pir| AF1348 competence negative regulator MecA	3.32	0.0047
	*ctsR*	SACOL0567	pir | D83662 transcription repressor of class III stress genes CtsR	3.16	0.0026
Biofilm formation	*isdC*	SACOL1141	gb | AAL33767.1 hypothetical protein SirD	3.37	0.0020
	*vwb*	SACOL0587	ref | NP_645583.1 truncated secreted von Willebrand factor-binding protein VWbp	3.36	0.0061
Cell wall-Surface	*capC*	SACOL0138	pir| C89776 capsular polysaccharide synthesis enzyme CapSC	3.33	0.0033
Antigen - Toxin - Virulence	*isaB*	SACOL2660	pir| F90071 immunodominant antigen B	3.30	0.0040
General functions [transporters, DNA-RNA, general]	*sceD*	SACOL2088	ref | NP_375203.l| hypothetical protein, similartoSceD precursor	3.36	0.0046
**(B)**					
Transcription regulators	*argR*	SACOL1565	ref | NP_692796.1arginine repressor (arginine metabolism regulator)	3.36	0.0021
	*gntR*	not found	ref | NP_656480.1 HTH_GNTR, helix_turn_helix gluconate operon transcriptional repressor	3.34	0.0055
	*lysR*	SACOL2555	ref| NP_242968.1transcriptional activator of the glutamate synthase operon (LysR family)	3.33	0.0029
	*ssrA*	SACOL1535	ref | NP_522722.1 Probable two-component response regulator transcription regulator I	3.33	0.0008
	*lytR*	SACOL2302	pir | F84108 attenuator for lytABC and lytR expression LytR	3.31	0.0002
Biofilm formation	*tpgX*	SACOL2365	ref | NP_375481.l| hypothetical protein, similartoTpgX protein	4.14	0.0323
	*emp*	SACOL0858	emb | CAB759S4.11 extracellular matrix and plasma binding protein	3.35	0.0056
	*ssaA*	SACOL0270	ref | NP_373516.l| hypothetical protein, similarto secretory antigen precursor SsaA	3.30	0.0020
Cell wall-Surface	*atl/lytD*	SACOL1062	ref| NP_391459.1N-acetylglucosaminidase (major autolysin) (CWBP90)	3.25	0.0047
	*capB*	SACOL0137	ref | NP_370674.1 capsular polysaccharide synthesis enzyme Cap5B	3.24	0.0039
Drug resistance	*femA*	SACOL1410	gb | AAC69631.1 factor essential for methicillin resistance FEMA	4.12	0.0356
	*sepA*	SACOL2158	dbj | BAB83937.11 SepA multidrug resistance efflux pump	3.38	0.0023
General functions [transporters, DNA-RNA, general]	*ybhK*	SACOL0831	pir| B90736 probable structural protein	4.08	0.0340
	–	not found	ref | NP_337929.l| phosphate transport system regulator PhoU-related protein	3.35	0.0016
	*mdlB*	SACOL2430	502776-1 Predicted CDS, ABC transporter with ABC transporter transmembrane region family	3.24	0.0075
	*sufB*	SACOL0918	ref | NP_3498S3.1 Iron-regulated ABC-type transporter membrane component (SufB)	3.22	0.0001

*The genes have been ranked according to their log_2_ fold change (pH9/pH7) value in each functional category.*

**TABLE 2:** Significantly upregulated genes at pH 5 in *S. aureus* COL biofilm-associated **(A)** and planktonic cells **(B)** (*p* < 0.05).

**Function**	**Gene name**	**Gene number (in *S*. *aureus* COL)**	**Gene annotation**	**Log_2_ fold change**	***p*-Value**
**(A)**					
Transcription regulators	*cymR*	SACOL16S1	ref | NP_464509.1 Weakly similar to two-component response regulator	3.25	0.0316
Biofilm formation	*mapW*	SACOL09S5	ref | NP_374103.l| Hypothetical protein, similar to cell surface protein Map-W	3.23	0.0149
	*efb*	SACOL1168	pir| DS9852 Fibrinogen-binding protein A, clumping factor	3.21	0.0048
	*vwb*	SACOL0857	ref | NP_645583.1 Truncated secreted von Willebrand factor-binding protein VWbp	3.16	0.0182
Cell wall-Surface	*capC*	SACOL26S5	pir| C89776 Capsular polysaccharide synthesis enzyme CapSC	3.28	0.0092
	*pgcA*	SACOL2501	ref | NP_109754.1 Two functions are possible, phosphomannomutase orphosphoglucomutase	3.20	0.0032
	*ebsB*	SACOL1471	ref| NP_374547-l| Hypothetical protein, similarto cell wall enzyme EbsB	3.12	0.0223
General functions [transporters, DNA-RNA, general]	*yozE-* like	SACOL2556	NP_375655.1 hypothetical protein	3.28	0.0127
	-	SACOL0366	ref | NP_646219-lTerminase small subunit	3.21	0.0029
	*metN1*	SACOL0504	pir| A!0131Methionine import ATP-binding protein MetNl	3.20	0.0028
	*hsdM*	SACOL0476	dbj | BAB41620.1/probale type I site-specific deoxyribonuclease Lldl chain hsdM	3.20	0.0030
**(B)**					
Transcription regulators	*bglG*	SACOL0228	pir| G97906 Transcription antiterminator BglG family BglG	3.25	0.0028
	*waIR*	SACOL0019	ref | NP_519473-1 Probable two-component response regulator transcription regulator protein	3.22	0.0050
	*rpoB*	SACOL05S8	sp| P4776S RPOB_STAAU DNA-directed RNA polymerase beta chain	3.21	0.0010
	–	SACOL0420	ref| NP_388770.1 Predicted transcriptional regulator	3.20	0.0356
	*mgrA*	SACOL0746	gb | AAK62673.1 Transcriptional regulator MgrA	3.19	0.0008
Cell wall-Surface	*tarS*	SACOL0243	ref| NP_346205-lGlycosyl transferase, family 2:glycosyl transferase family 8	3.28	0.0014
	*capB*	SACOL0137	ref | NP_370674.1 Capsular polysaccharide synthesis enzyme Cap5B	3.14	0.0046
	*copC*	SACOL0138	pir | C89776 Capsular polysaccharide synthesis enzyme Cap8C	3.07	0.0235
Drug resistance	*femA*	SACOL1410	gb | AAC69631.1 Factor essential for methicillin resistance FemA	4.12	0.0354
	*pis*	SACOL0050	sp | P80544 MRSP_STAAU Methicillin-resistant surface protein precursor	3.10	0.0026
General functions [transporters, DNA-RNA, general]	–	not found	ref | NP_656125-lMS_channel, Mechanosensitive ion channel	3.50	0.0005
	*recF*	SACOL0004	sp | Q9RVE01 RECF_DEIRA DNA replication and repair protein RecF	3.34	0.0058
	*mdtB*	SACOL2430	ref | NP_502776.1 Predicted CDS, ABC transporter with ABC transporter transmembrane region family member	3.20	0.0113
	*sasA*	SACOL2676	gb | AAL5S470.11 AF459093_1 Serine-threonine rich antigen	3.17	0.0055
	*hchA*	SACOL0597	dbj | BAA15794.1 H-NS-repressed protein, 30K	2.99	0.0074
	*hup*	SACOL1513	sp | P091681 OGT_ECOLI DNA-binding protein HU	2.74	0.0154

*The genes have been ranked according to their log_2_ fold change (pH5/pH7) value in each functional category.*

In planktonic cells growing at pH 9, sixteen genes were over-expressed, with log_2_ fold-change ratios between 3.22–4.14 and a *p*-value < 0.0356 (Table 1B). They included genes encoding five transcription regulators, one protein associated with drug resistance (the essential factor for methicillin resistance, FEMA) and nine other proteins with various functions. The transcription factors included an attenuator for lytABC and LytR expression (LytR), a two-component response regulator (SrrA), the repressors for arginine (ArgR) and gluconate biosynthesis (GntR), and an activator of glutamate synthase (LysR/CidR). The other up-regulated genes coded for two ABC transporters with transmembrane functions (MdlB and an iron regulating, SufB), a phosphate transport system regulator (PhoU-related protein), a major autolysin (Atl/LytD), a hypothetical; protein similar to secretory antigen precursor SsaA (LysM), the capsule biosynthetic enzyme CapB, a cell wall-related enzyme SepA, an extracellular matrix and plasma binding protein (Emp), and two probable proteins (similar to TpgX protein and a structural protein, similar to YbhK).

A similar number of genes (16) were also over-expressed in planktonic cells growing at pH5, with log_2_ fold-change ratios between 2.74–4.12 and a *p*-value < 0.0354 (Table 2B). They included three genes associated with DNA or RNA functions (the replication and repair protein RecF, the DNA-binding protein Hup, and the DNA-directed RNA polymerase beta chain, RpoB), four gene products involved in regulation of transcription (the transcription regulators MgrA, WalR, SACOL0420, and the antiterminator BglG), two capsular biosynthesis enzymes (CapB and CapC), a serine-threonine rich antigen (SasA), two genes associated with methicillin resistance (the essential factor for methicillin resistance, FEMA, and the methicillin-resistance surface protein precursor, Pls), an ABC transporter (MdlB), a glycosyl transferase involved in colanic acid biosynthesis (TarS), and two factors with ill-defined function (a mechanosensitive ion channel protein and an H-NS-repressed protein HchA).

In BF cells growing at pH9, the down-regulated genes coded for two transcriptional regulators (AirR and the two-component response-regulatorNreB), a phosphate transport system regulator (PhoU-related protein) and a cell surface protein (MapW). All had log_2_ fold change ratios between −3.24 and −3.34 and *p*-values < 0.0042 (Table 3A). At acidic conditions (pH5) 18 genes were down-regulated in BF-forming cells. Two of these were as at pH9 (AirR and SACOL0420), three coded for transcriptional regulators (HutR, SACOL2517, and BglG), two were associated with toxin production (Ssl1 and Ssl11) and another three with cell surface (EpiG, MapW and elastin binding protein EbsS). Down-regulated were also the signal peptide precursor AgrD, the cell division FtsK, the RNA polymerase beta subunit RpoB, the translation elongation factor TufA, the probable translational initiation factor (InfB), a stress response DNA-binding protein Dps, a peptidoglycan hydrolase (LytM) and interestingly enough the plasmid mobilizing protein Mob. All had log2 fold change ratios between −2.37 and −4.05 and *p*-values < 0.0365 (Table 4A).

**TABLE 3:** Significantly down-regulated genes at pH 9 in *S. aureus* COL biofilm-associated **(A)** and planktonic cells **(B)** (*p* < 0.05).

**Function**	**Gene name**	**Gene number (in S. *aureus* COL)**	**Gene annotation**	**log_2_ fold-change**	***p*-Value**
**(A)**					
Transcription regulators	*airR*	SACOL1905	ref | NP_388770.1 Predicted transcriptional regulator	−3.34	0.0042
	*degU*	SACOL2389	ref| NP_714049.1 Two-component response regulator transcriptional regulator protein	−3.32	0.0015
	*phoU-like*	not found	ref | NP_337929.l| Phosphate transport system regulator PhoU-related protein	−3.28	0.0023
Biofilm formation	*mapW*	SACOL0985	emb | CAB51S07.1 Cell surface protein Map-W	−3.24	0.0014
**(B)**					
Transcription regulators	*sarA*	SACOL0672	gb | AAM74164.11 AF515775_2 Staphylococcal accessory regulator variant	−3.43	0.0317
	*agrB*	SACOL2023	ref | NP_469388.1 Similar to Staphylococcus two-component sensor histidine kinase AgrB	−3.38	0.0187
Biofilm-related proteins	*mntC*	SACOL0638	NP_720653 a surface adhesion precursor	−3.34	0.0042
Cell wall-related enzymes	*murAB*	SACOL2116	sp | Q99SD4| MUA2_STAAM UDP-N-acetylglucosamine 1-carboxyvinyltransferase 2 (Enoylpyruvate transferase 2)	−3.33	0.0068
	*lytM*	SACOL0263	pir| F89791 Peptidoglycan hydrolase	−3.33	0.0109
Antigen - Toxin - Virulence	*sek*	SACOL0336	gb | AAC2S96S.1 Staphylococcal enterotoxin K	−3.45	0.0036
Drug resistance	*mdeA/emrB*	SACOL2413	ref| NP_375526.l| MFS transporter; drug resistance transporter EmrB/QacA subfamily	−3.34	0.0042
General functions [transporters, DNA-RNA, general]	*yozE-* like	SACOL2556	ref | NP_375656-11 hypothetical protein, similarto secretory antigen precursorSsaA	−4.19	0.0461
	*parC*	SACOL1390	ref | NP_758033.1 DNA topoisomerase IV subunit A	−3.33	0.0065
	–	not found	ref | NP_720897.1 Putative cell division protein	−3.33	0.0068
	*maoC*	not found	ref | NP_280923.1 Monoamine oxidase regulatory-like	−3.31	0.0095

*The genes have been ranked according to their log_2_ fold change (pH5/pH7) value in each functional category.*

**TABLE 4:** Significantly down-regulated genes at pH 5 in *S. aureus* COL biofilm-associated **(A)** and planktonic cells **(B)** (*p* < 0.05).

**Function**	**Gene name**	**Gene number (in *S. aureus* COL)**	**Gene annotation**	**log2 fold-change**	***p*-Value**
**(A)**					
Transcription regulators	–	SACOL0420	ref | NP_388770.1 Predicted transcriptional regulator	−3.57	0.0035
	*bglG*	SACOL0228	pir| G97906 Transcription antiterminator BgIG family BgIG	−3.42	0.0057
	*hutR*	SACOL2325	ref | NP_52065S.l Probable transcription regulator transcription regulator protein	−3.40	0.0059
	–	SACOL2517	ref | NP_665411.1 Putative transcriptional activator regulator protein	−3.19	0.0031
	*rpoB*	SACOL0588	sp| P47768| RPOB_STAAU DNA-directed RNA polymerase beta chain	−3.17	0.0087
	*agrD*	SACOL2024	gb | AAF72185-11AF255950J. AgrD signal peptide precursor	−3.17	0.0116
	*airR*	SACOL1905	ref | NP_714049.1 Two-component response regulator transcriptional regulator protein	−3.11	0.0028
	*dps*	SACOL2131	ref | NP_45980S.l| Stress response DNA-binding protein; starvation induced resistance to H202	−3.05	0.0107
Biofilm formation	*mapW*	SACOL0985	emb | CAB51807.1 Cell surface protein Map-W	−3.40	0.0023
	*epiG*	SACOL1871	dbj | 8AB95623.11 Epidermin immunity protein F	−3.26	0.0036
Antigen - Toxin - Virulence	*Ssl11*	SACOL0478	pir| C89808Exotoxinl5	−3.49	0.0006
	*Ssl1*	SACOL0468	pir| G89806Exotoxin6	−3.37	0.0018
General functions [transporters, DNA-RNA, general]	*infB*	SACOL1285	emb | CAD55362.1 Probable translational initiation factor; putative translation initiation factor lF-2(fragment)	−4.05	0.0365
	*ftsK*	SACOL1295	ref | NP_459936.1 Cell division protein, required for cell division and chromosome partitioning	−3.41	0.0022
	*lytM*	SACOL0243	pir| F89789 Cell division and morphogenesis-related protein	−3.37	0.0051
	*tufA*	SACOL0594	pir| T44381 Translation elongation factorTu (EF-Tu) TufA	−3.11	0.0167
	*mob*	SACOLRS00015	gb| AAA93296.1 Mobilization (Mob):recombination (Pre) protein	−2.37	0.0272
	*ebsS*	SACOL1522	gb | AAC441352 Cell surface elastin binding protein	−3.19	0.0085
**(B)**					
Transcription regulators	*hfq*	SACOL1324	sp | P255211 HFQ_ECOLI Hfq protein (Host factor-! protein) (HF-I) (HF-1)	−3.53	0.0024
	*sarA*	SACOL0672	gb | AAM74164.11 AF515775_2 Staphylococcal accessory regulator variant	−3.39	0.0308
Biofilm formation	*scc*	SACOL1169	ref | NP_374275.11 Hypothetical protein, similartofibrinogen-binding protein	−3.47	0.0163
	*mapW*	SACOL0985	emb | CAB51S07.1 Cell surface protein Map-W	−3.43	0.0019
Antigen - Toxin - Virulence	*Ssl14*	SACOU1S0	ref | NP_374284.l| Hypothetical protein, similarto exotoxin 3	−3.49	0.0139
	*isaB*	SACOL2660	pir| F90071 Immunodominant antigen B	−3.27	0.0066
General functions [transporters, DNA-RNA, general]	*maoC*	SACOL0032	ref | NP_280923.1 Monoamine oxidase regulatory-like; MaoCl	−3.49	0.0097
	*parC*	SACOL1390	ref | NP_758033.1 DNA topoisomerase IV subunit A	−3.44	0.0050
	*metN2*	SACOL0504	pir | G71363 Probable amino acid ABC transporter, ATP-binding protein (abc)	−3.44	0.0066
	*mntC*	SACOL0683	ref | NP_720653.1 Putative ABC transporter, metal binding lipoprotein; surface adhesin precursor; lipoprotein receptor Lral	−3.42	0.0027
	*prsA*	SACOL1897	sp | Q92H911 PLP_RICCN Parvulin-like PPIase precursor (Peptidyl-prolyl *cis*-*trans* isomerase Pip)	−3.23	0.0093
	–	not found	ref | NP_437451.1 Conserved putative membrane protein, possibly a permease	−3.05	0.0129

*The genes have been ranked according to their log_2_ fold change (pH5/pH7) value in each functional category.*

For the planktonic cells under alkaline conditions, 11 genes were clearly down-regulated (log2 fold change ratios between −3.31 and −3.38 and all *p*-values < 0.0317, Table 3B). These included the staphylococcal accessory regulator SarA, the two-component sensor of histidine kinase AgrB and the staphylococcal enterotoxin K (Sek). Also, a monoamine oxidase (MaoC1), a DNA topoisomerase IV subunit A (ParC), a surface adhesin precursor (MntC), a peptidoglycan hydrolase (LytM), a putative UDP-N-acetylglucosamine 1-carboxyvinyltransferase 2 (MurAB), a hypothetical multidrug resistance protein (MdeA/EmrB), a putative cell division protein (NP_720653.1) and a multispecies conserved protein (YozE-like, SACOL2556).

Similarly, for the planktonic cells in acidic conditions 12 genes were down-regulated (log2 fold change ratios between −3.05 and −3.53 and *p*-values < 0.0308, Table 4B). Genes coding for MaoC1, ParC and MntC were down-regulated as under alkaline conditions, and in addition the amino acid ABC transporter (MetN2), the host factor protein (Hfq), the staphylococcal accessory regulator SarA, exotoxin 3 (Ssl14), fibrinogen-binding protein (Scc), immunodominant antigen B (IsaB), cell surface protein (MapW), a parvulin-like PPIase precursor (PrsA) and a putative permease (NP_437451.1).

**FIGURE 2:**
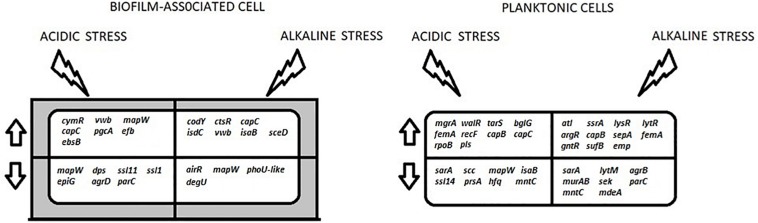
A summary of the key differentially-expressed genes that are discussed in this study. These genes are involved in the processes of transcription regulation (*ctsR*, *argR*, *lysR*, *ssrA*, *lytR*, *cymR*, *bglG*, *walR*, *rpoB*, *mgrA*, *airR*, *degU*, *sarA*, *agrD*, *phoU-like*, *agrB*, *dps*, *hfq*), cell wall biosynthesis (*capB*, *capC*, *tarS*, *pgcA*, *ebsB*), BF formation (*mapW*, *efb*, *isdC*, *vwb*, *trgX*, *emp*, *ssaA*, *mntC*, *epiG*, *scc*), autolysis (*atl*, *murAB*, *lytM*), virulence (*isaB*, *ssl11*, *ssll1*, *ssl14*, *sek*) and antibiotic resistance (*femA*, *sepA*, *pls*, *mdeA*). The arrows indicate up- and down-regulation. The shaded area around the BF-associated cell represents the extracellular matrix.

Figure 2 summarizes the gene expression changes of genes associated with BF formation that were observed in this study, under different conditions.

## Discussion

Staphylococci are commensal bacteria and the most prevalent on human skin and mucous. Due to their ability to freely form BFs and, as such, to persist on indwelling medical devices, they are the most frequent culprits of nosocomial infections and cause severe problems to patients who have undergone surgical operations (Trindade et al., 2017). The discovery that in BF formed by several pathogenic bacteria, including *S. aureus*, the BF-associated bacteria are up to 1,000-fold more tolerant to antibiotics than their genetically identical planktonic cells, attracted the interest of many scientists. As a result, research on the molecular regulatory mechanisms that influence BF formation in *S. aureus* has intensified during the past two decades (Pratten et al., 2001; Beenken et al., 2004; Resch et al., 2005; Archer et al., 2011; Periasamy et al., 2012; Guilhen et al., 2017; Otto, 2018). The first two microarray studies on *S. aureus* BF formation were performed in a murine model of catheter-based BF (Beenken et al., 2004) and dialysis membranes laid on agar plates (Resch et al., 2005). In both cases over-expression of large numbers of genes coding for cell wall-associated proteins, transport proteins, secreted proteins, enzymes and transcription regulators was observed in BF cells. These findings were later verified by a proteomic analysis of BF cells and correlation with the transcriptomic profiles of *S. aureus* (Resch et al., 2006). Similarly, transcriptomic studies that focused on *S. aureus* in liquid cultures have identified over-expression of a variety of genes, when the cells grew under acidic or alkaline conditions. For example, under acidic conditions *cap5B*, *cap8C*, other capsule biosynthesis genes, *isaA*, *ssaA*, an autolysin gene and *fnb*A, *ctsR*, *phoP* were up-regulated (Weinrick et al., 2004; Bore et al., 2007), whereas *fnbB*, Na^+^/H^+^ antiporters, and *lytR* were down-regulated (Anderson et al., 2010; Rode et al., 2010). Similarly, Na^+^/H^+^ antiporter and Cap5 enzyme genes were over-expressed under alkaline conditions (Anderson et al., 2010). The fact that most of these genes were also identified as differentially expressed in our study confirms that they are indeed important when the cells are exposed to pH-associated stress. Overall, under the same pH conditions, more genes were up- or down-regulated in planktonic cells than in BF cells (Tables 1–4).

Bacteria can survive and even thrive under harsh environmental conditions often due to their capacity to form BFs. Abundance or depletion of nutrients, carbon and nitrogen sources, presence or absence of oxygen, electron acceptors, acidic or alkaline conditions, etc., proved to be major environmental stress factors that induce or prevent BF formation in *S. aureus* (Weinrick et al., 2004; Boles and Horswill, 2008; Anderson et al., 2010; Rode et al., 2010; Mashruwala et al., 2017). The ability of bacteria to sense chemical and physical characteristics of their surroundings and adjust gene expression require mechanisms that take decisions in response to environmental condition changes, and these are mainly based on protein–DNA interactions defined by transcriptional factors (TFs) and their targets around promoters. In *S. aureus* 135 TFs and sigma factors of various family groups have been identified with only half of them experimentally characterized to date (Ibarra et al., 2013). Our results show that the best characterized global transcription regulators like Agr, SarA, AirR, CodY, CtsR, MgrA, LysR, WalR, SrrA, along with several other less studied TFs, like CidR, BglG, ArgR, LytR, CymR, HutR, Hup, InfB, NreB, Dps, as well as some of putative function like the GntR family SACOL2516, the Xre family SACOL0420, the Mer family SACOL2517, all of which are associated with BF formation, were differentially expressed under acidic or alkaline conditions. Of the above TFs, pivotal role play those that are also members of two-component systems (TCS), signal transduction mechanisms utilized by most bacteria to monitor and respond to environmental stimuli. TCSs are composed by a membrane protein sensor (histidine kinase) and a response regulator which receives the information from the kinase and brings about the relevant response. Out of the 16 known TCSs in *S. aureus* only WalKR is essential (Dubrac et al., 2007) and as our results show, apart from WalKR and AgrBDCA five more TCSs were differentially expressed, i.e., three associated with oxygen availability in the media (SrrAB, AirRS and NreAB), one involved in autolysis (LytRS), and one sensing K^+^ limitation or salt stress (KdpDE).

In association with its involvement in BF formation, the *agr* Quorum-Sensing (QS) system of *S. aureus*, is the best studied in the bacterium. AgrB and AgrD act to generate the auto-inducing peptide AIP (QS molecule) which, after reaching an extracellular threshold concentration, stimulates activation of the TCS regulatory system AgrC (sensor) and AgrA (regulator). Under normal growth conditions, during BF formation, the Agr QS system is repressed to stop the expression of *S. aureus* colonization factors (Novick, 2003) and it gets activated mostly in the bacteria of the outer BF layers leading to the dispersal (Thoendel et al., 2011). In this respect, the down-regulation of AgrD, the peptide precursor of AIP, in BF cells at pH5 is explained by the effort of cells to promote BF formation as a defense against the acidic conditions. Similarly, the down-regulation of AgrB, the membrane protein which secrets the AIP product, is also expected in planktonic cells at pH9, in the same way as shown in *S. aureus* strain UAMS-1 where *agr*B expression was up-regulated in early growth stages and completely shut off at later stages (Grande et al., 2014). The accessory gene regulator (*agr*A) and the staphylococcal accessory regulator (*sar*A) have opposing roles in *S. aureus* BF formation and exert pleiotropic effects on the expression of molecules responsible for binding to different surfaces, controlling large numbers of target genes involved in virulence, autolysis, stress responses and metabolic processes (Pratten et al., 2001; Bischoff et al., 2004). The primary regulatory role of SarA is to repress the production of extracellular nucleolytic and proteolytic enzymes in early BF formation and once BF have developed and matured, *agr* expression leads to up-regulation of a number of virulence factors (Luong and Lee, 2002). Therefore, its down-regulation in planktonic cells at pH9 should have been accompanied by the induction of virulence factors, which is not the case (Tables 1, 3). However, although our results contradict with those obtained from BF cells grown under normal growth conditions, they are in full agreement with studies showing that *agr* expression in BF development strongly depends on environmental conditions and clearly underline the influence of the alkaline environment (Yarwood et al., 2004). Bearing also in mind that LytM belongs to the staphylin-type peptidase family and SsaA is a staphyloxanthin biosynthesis protein, their down-regulation in planktonic cells at pH9 along with SarA and AgrB is easily understood. MgrA, an important member of the SarA family, was also up-regulated in planktonic cells at pH5, along with WalR and TarS. MgrA is a global regulator that modulates the expression of 5–10% of the *S. aureus* genome, controls autolysis, virulence, a number of large surface proteins and most importantly, activates the *agr* system, thus repressing BF formation in *S. aureus* (Luong et al., 2006; Crosby et al., 2016). Known to form in combination with the TCS ArlRS a regulatory cascade and regulate BF formation (Crosby et al., 2016), *mgr*A’s over-expression was more or less expected in the absence of ArlRS activity. However, this is not the case for WalR, the response regulator of the two-component system (TCS) WalKR (also known as YycGF), which positively controls BF formation in *S. aureus* and many genes involved in cell wall degradation, like *atl*A, *lyt*M, *isaA*, *sceD*, *ssaA*, and four *ssaA*-related genes (Dubrac et al., 2007). This TCS positively regulates major virulence genes involved in host matrix interactions (like *efb*, *emp*, *fnbA* and *fnbB*), oxidative stress resistance and vancomycin resistance (Delaune et al., 2012). Since none of the above-mentioned genes was up-regulated under acidic conditions, the most likely explanation is the loss of proper WalR function due to conformational changes that take place in its functional domains, i.e., the DNA-binding domain and the phosphorylation reception domain, under these pH conditions. This may happen by an alteration of the phosphorylation state of the conserved histidine residue in the cytoplasmic kinase, therefore preventing the response regulator to bind specifically on its DNA targets.

Two TCSs associated with oxygen availability in the media, AirRS and NreBC, were down-regulated in BF cells at pH9, along with a putative phosphate transport system regulator, PhoU-like, which encodes a probable transcriptional regulatory protein homologous to PhoU proteins involved in the down-regulation of phosphate uptake. AirRis the DNA-binding response regulator of the TCS AirSR that senses oxygen and redox changes, and also regulates pathways for nitrate respiration and lactose catabolism, as well as virulence factors (Sun et al., 2012). In a concerted action with SrrAB they reduce *agr* expression under conditions of low oxygen availability and are required for BF formation (Yarwood et al., 2001; Ulrich et al., 2007). AirR was also down-regulated at pH5, underlining the adverse effects of pH stress on its function and verifying its importance for BF formation. Knowing that AirR’s activity state is determined by oxidation of an Fe-S cluster present in AirS (Sun et al., 2012), it is feasible to assume that the prolonged exposure of cells to alkaline or acidic conditions could have destroyed this cluster in AirS, -which is essential for its kinase activity-, and thus hamper DNA binding activity of AirR and allow BF formation. A similar dissociation of Fe-S at pH9 would have also been the explanation for the down-regulation of NreB, the sensor histidine kinase protein of the TCS, a cytoplasmic protein containing four conserved cysteine residues that together comprise a Fe-S cluster (Kamps et al., 2004). In regard to the putative PhoU-like phosphate transport regulator it should be pointed out that recently the function of two *phoU* genes has been experimentally determined in *S. epidermidis* proving that only one of these is an important regulator of BF formation and drug tolerance (Wang et al., 2017). Although sequence similarity of the PhoU-like COL gene with either of these genes is low, its up-regulation in planktonic cells grown at alkaline conditions signifies the important role of this putative protein in response to pH stress.

In BF cells growing at acidic conditions, we have recorded a moderate up-regulation of KdpD, regulator of the kdpDE TCS (Supplementary Table S1). This TCS responds to K^+^ limitation and salt concentrations and it is up-regulated by *agr* through repressing *rot* translation (Xue et al., 2011). Its up-regulation in both BF and planktonic cells at pH5 is regarded as an indication of no K^+^ limitation in the media and also confirms its dependence on the *agr* system. Under the same growth conditions CymR, MetN2, and HsdM were also up-regulated in BF cells. CymR is the master regulator of cysteine metabolism in *S. aureus* known to control host sulfur source utilization and also play a role in BF formation (Soutourina et al., 2009). MetN2, is an ABC transporter ATP-binding protein (member of the three-gene operon *met*N2 > SACOL0505 > 0506), involved in metabolism. Their up-regulation is in full agreement with results which show that CymR under stress requires more cysteine and therefore up-regulates several target genes including *metNPQ* (Chang et al., 2006). HsdM, is the site specific restriction-modification enzyme used in the bacterial defense system against foreign DNA. Therefore, its up-regulation in BF cells may be due to the bacterial sensing of excess extracellular DNA produced by the lysis of outer layer cells and the strengthening of matrix that takes place in the formed BF.

The staphylococcal respiratory response TCS, SrrAB, is critical for anaerobic growth of *S. aureus* as the membrane component SrrB senses oxygen limitation and signals the cytoplasmic SrrA to repress transcription of the accessory gene regulator *agr* (Yarwood et al., 2001). Under normal conditions SrrA binds to its own promoter (autoregulation) as well as to numerous other promoters as its complex regulatory role has been revealed by a recent microarray analysis which shows that a Δ*srr*A mutation affects the transcription of 230 genes in normal growth conditions, and 51 under decreased oxygen (Wu et al., 2015). Impaired respiration leads to increased cell lysis via increased expression of *atl*A, resulting also to the release of DNA, cytosolic proteins and BF formation (Mashruwala et al., 2017). This is in full agreement with our observation that in planktonic cells grown at pH9, LytR, the transcriptional regulator of the *S. aureus* TCS system LytRS and affector of murein hydrolase activity, and AtlA, a murein hydrolase, were up-regulated. Due to their lytic activities both proteins are linked with cell wall synthesis, autolysis and release of genomic DNA that eventually becomes an important part of the BF matrix thus positively affecting BF formation (Lehman et al., 2015). The concerted up-regulation of the above genes along with genes involved in general metabolism like *pho*U, *arg*R (arginine metabolism repressor), *lys*S (transcriptional activator of the glutamate synthase operon), *gnt*R (gluconate operon transcriptional regulator), *ybh*K (a putative phospho-L-lactate transferase-like protein) and *suf*B (an ABC-type transporter membrane component), strongly indicate that they contribute to cell lysis and release of genomic DNA. In particular the strong influence of arginine on polysaccharide intercellular adhesin synthesis and BF formation that has been recorded in *S. aureus* (Zhu et al., 2007) the up-regulation of ArgR is expected to withhold BF formation in planktonic cells. The glutamate metabolism activator (LysR, also known as CidR in other strains) could also be involved in stress response as poly-γ-DL-glutamic acid is a virulence factor that protects *S. epidermidis* against high salt concentrations and additionally mediates resistance to antimicrobial peptides and phagocytosis (Fey and Olson, 2010). Similarly, as the iron-regulated transporter SufB is associated with resistance to oxidative stress it can act as a protection mechanism due to its non-specific DNA binding ability (Mashruwala et al., 2015). Proteins SufB and SufU synthesize inorganic cofactors called iron-sulfur (Fe-S) clusters, which are required for functional Fe-S proteins. Mutant *S. aureus* cells that are unable to transfer iron-sulfur clusters have impaired pathogenicity and are more sensitive to stress due to endogenous reactive oxygen species (ROS) which leads to DNA damage, and exogenously supplied ROS and reactive nitrogen species. Thus, as the planktonic cells grown under alkaline conditions are in late exponential phase, the over-expression of lytic enzymes can be explained as the preparation of the cells to form BF as soon as the conditions become favorable (i.e., as soon as they reach post-exponential phase and there is a clear nutrient depravation or when the pH drops to pH7, which happens after 2–3 days). Interestingly enough, none of the known for *S. aureus* lytic enzymes was up-regulated in planktonic cells under acidic conditions, underlining the strong repression of the corresponding genes.

The major TRs, CodY and CtsR were over-expressed in BFs under alkaline conditions. The importance of CodY as a global regulator of *S. aureus* has been revealed by a genome-wide analysis using DNaseI foot-printing assays which has shown that it has more than 200 direct gene targets (Majerczyk et al., 2010). As a DNA-binding protein it interacts directly with chromosomal DNA containing a conserved sequence stem-loop motif and affects BF formation both positively and negatively depending on the strain (Brinsmade, 2017). It has been suggested that under normal growth conditions it suppresses BF formation in methicillin-resistant strains (such as the MRSA strain used in this study) and promotes BF formation in methicillin-susceptible strains (Atwood et al., 2015). This contradiction with our findings can be attributed either to the different strains used or to the adverse effect of alkaline conditions on the bacterium. Nevertheless, the known repression of virulence gene expression by CodY (Waters et al., 2016) is also recorded in our results. CtsR is a global transcriptional regulator of protein quality control which under normal conditions is active as a repressor binding to its cognate DNA operator sequences. In *S. aureus* it is the first of a four-gene operon (*cts*R, SACL0568, SACL0569, *clp*C) and its protein acts as the negative regulator of the Class III family of heat shock genes *clp*C, *clp*B and *clp*P; the latter acting as a global regulator on regulons involved in virulence, oxidative stress response, autolysis and DNA repair (Michel et al., 2006). Under exposure to stress *cts*R losses its ability to bind DNA because of conformational changes and that leads to an un-induced transcription of target genes (Derre et al., 1999). Thus, the over-expression of *cts*R in BF cells grown under alkaline conditions indicates that the gene exerts its negative control on *clp* shock genes, including the ClpC protease. This comes to agreement with the over-expression of the negative regulator of competence MecA that we observed under the same conditions, a protein which binds to ClpC and prevents proteolysis (Tian et al., 2013). The need for autolysis in order to maintain the BF and enhance the rigidity of extracellular matrix by the release of eDNA is compensated at alkaline conditions by the over-expression of the lytic transglycosylase SceD, which is known to promote BF formation and is essential for nasal colonization in cotton rats (Stapleton et al., 2007), and the secreted immunodominant surface protein IsaB, which has the ability to bind eDNA and stabilize the extracellular matrix (Gibert et al., 2014).

Under acidic conditions along with AirR and AgrD several TFs, namely HutR, BglG, InfB, Dps, SACOL0420 and SACOL2517 were also down-regulation in BF cells together with proteins RpoB, FtsK and TufA, of general cell maintenance functions. HutR is a transcriptional regulator of the LysR family, which is the most common type of bacterial DNA-binding proteins, acting as either activators or repressors of gene expression and considered as a putative repressor of the histidine utilization operon (Ibarra et al., 2013). The BglG family transcriptional anti-terminators are DNA-binding proteins that regulate the expression of bacterial genes and operons, whose products are required for utilization of phosphoenolpyruvate:sugar phosphotransferase system carbohydrates (Fux et al., 2004). In *S. aureus* COL the *bgl*G gene that was down-regulated was SACOL0228, the mannitol operon transcriptional antiterminator. It is noted that there are three more *bgl*G loci in *S. aureus* COL genome with highly similar protein sequences (SACOL0403 and SACOL2147, mannitol operon transcriptional anti-terminators; and SACOL2662, activator of the mannose operon). SACOL2517 is a putative transcriptional regulator of the MerR family, contains a HTH domain and shows extended similarity with the gluconate operon transcription regulator GntR. InfB is the translation initiation factor IF-2, one of the essential components for the initiation of protein synthesis. It protects formyl-methionyl-tRNA from hydrolysis and promotes its binding to the 30S ribosomal subunits. Dps is a DNA-binding stress response protein for which very little is known about its exact function in *S. aureus.* In *E. coli* it protects DNA in a non-specific way from acid-induced damage (Jeong et al., 2008). However, the same study shows that even in the presence of Dps, protein RecA is needed for the repair of acid-induced DNA damage. In addition to the above, *rpo*B, the DNA directed RNA polymerase βchain coding gene, *fts*K, the gene coding for a protein required for cell division and chromosome partition and the translational elongation factor *tuf*A involved in peptide chain formation were also down-regulated in BF cells under acidic conditions. Taking into consideration the functions of all the above down-regulated TFs and proteins, we may assume that in order to maintain the BF, cells at pH5 drastically reduce metabolic activities, thus preventing cell lysis and dispersal which would require the protection of DNA. In support of this hypothesis may also be considered the down-regulation of *mob*, which codes for a protein causing the mobilization of *S. aureus* COL plasmid pT181, hence it prevents the potential horizontal gene transfer of plasmid carried antibiotic resistance genes. Finally, the down-regulation of SACOL0420 in BF cells in contrast with its up-regulation in planktonic cells at pH5, indicates that this putative transcriptional regulator of the Xre family, may play an important role in BF formation. Its sequence analysis shows that it contains a signal transduction peptide, REC and HTH domains, and it is the first gene of an operon 0420 > 0421 > 0422 > 0424, which is putatively regulated by NreC. Therefore, since its true function is still unknown it certainly merits more attention in the future.

In the early stages of BF formation *S. aureus* cells are passively adsorbed on the material surface through electrostatic and hydrophobic interactions. Following the initial cell adhesion and formation of a monolayer, a cell to cell aggregation and accumulation in bacterial multilayered architecture is mediated by MSCRAMMs, proteins with differential binding specifications for host matrix components and all containing an LPXTG motif that allows them to anchor on surfaces (Patti et al., 1994; Stoodley et al., 2002). All these MSCRAMMs are covalently attached to cell wall peptidoglycan by the membrane-associated enzyme sortase that recognizes the LPXTG motif and their corresponding genes are controlled and up-regulated by the sigma B (Bischoff et al., 2004). Several transcriptomic studies with pathogenic isolates of *S. aureus* have shown that under normal pH conditions all these genes are up-regulated in BF and planktonic cells (Dunman et al., 2001; Resch et al., 2005; Lindsay et al., 2006; Wang et al., 2012). It is, therefore, interesting to note that in our study only two of these proteins were differentially expressed in BF cells under the pH stress conditions, i.e., the secreted VWF-binding protein (Vwb), which was up-regulated under both pH regimes, and EbsS, an elastin binding protein, the only adhesive *trans*-membrane MCRAMM that contains the pentapeptide motif NPQTN instead of LPXTG and was the only down-regulated MSCRAMM family protein at pH5. The two large surface associated glycoproteins, VWF and SasA, both mediating platelet adhesion at sites of endothelial damage were up-regulated in planktonic cells grown at pH5 (Tables 1, 2). The Von Willebrand factor (VWF) is a large, multimeric glycoprotein mediating platelet adhesion at sites of endothelial damage and Vwb interacts with VWF and the surface protein clumping factor A (ClfA), thus anchoring *S. aureus* to vascular endothelium under shear stress, enhancing its ability to cause tissue damage or systemic disease (Claes et al., 2017). It appears therefore, that in BF-associated cells Vwb not only can adhere on polystyrene surfaces under both alkaline and acidic conditions, but obviously plays a pivotal role in cell to cell aggregation and accumulation under these stress conditions. SasA, also known as Srap (serine-rich adhesin for platelets) mediates the direct binding of *S*. *aureus* to platelets and contributes to infective endocarditis is a less studied MSCRAMM protein which contains the LPXTG motif (Siboo et al., 2005). SasA has been found responsible for binding to gp340 -a factor that in the oral cavity induces salivary aggregation with bacteria and promotes *S. aureus* adhesion to tissues such as the teeth and mucosa- via the *N*-acetyl-neuraminic acid moiety (Kukita et al., 2013). Thus, its up-regulation is considered as an additional tool for binding on abiotic surface. The down-regulated at pH5 protein EbsS, due to its structure, is under different regulatory control as it is known that the constitutively expressed sortase SrtA is responsible for anchoring all LPXTG-containing surface proteins, whereas SrtB is specialized to carry out the specific iron-regulated cell wall sorting of a NPQTN signal proteins like EbsS and IsdC (Mazmanian et al., 2002).

Biofilm maturation starts when the intrinsic regulatory program of BF formation begins to produce the matrix, consisting of extracellular capsular polysaccharides, proteins and eDNA. Altogether these molecules organize cells in three-dimensional structures, separated by fluid channels which are vital in delivering nutrients into BF deeper layers, as well as to deliver auto-inducing peptides that sense population densities (QS) and are subsequently used to trigger dispersal of cells and virulence factors (Boles et al., 2010; Thoendel et al., 2011; Otto, 2013). Concerning cell wall enzymes, in accordance to what has been observed under normal pH conditions (Resch et al., 2006; Beenken et al., 2012) it is interesting to note that the capsular polysaccharide biosynthesis enzymes Cap8C and Cap5B were up-regulated at both pH regimes, the former in BF cells –as well as in planktonic cells at pH5- and the latter in planktonic cells (Tables 1, 2). Thus, apart from their established role in BF development, the up-regulation of Cap5B and Cap8C is most likely due to the effort of cells to repair their capsule and cell wall after damage caused by the alkaline or acidic environment. Knowing that in a previous study under mild acid conditions (pH5.5) both Cap8C and Cap5B were significantly up-regulated in BF cells (Weinrick et al., 2004), the lack of induction of Cap5B at slightly lower pH in our work possibly underlines the importance of small pH differences on gene expression in BFs. Facilitated by LytR-A-Psr family enzymes, Cap5B is known to covalently attach to the glycan strands of peptidoglycan (Chan et al., 2014), and this seems to be the case in planktonic cells growing at pH9 that showed up-regulation of both *lyt*R and *cap*5B (Table 1). It is pointed out that the over-production of type 8 capsular polysaccharides was previously found to augment *S. aureus* virulence, leading to longer persistence in the bloodstream, the liver, and the spleen of experimental mice (Luong and Lee, 2002), however, without influencing the pathogen’s susceptibility to vancomycin (Jansen et al., 2013). EbsB, a putative cell wall enzyme and PgcA, a phosphomannomutase/phosphoglucomutase family protein were also up-regulated in BF cells at pH5. EbsB contains a nucleic acid-binding motif, which may have an additional role either as a regulator or in association with eDNA. PgcA is involved in the biosynthesis of UDP-N-glucosamnine and a *pgc*A-transposon inactivated gene in *S. aureus* was shown to have drastically reduced methicillin resistance, although its *fem*A gene remained intact (Wu et al., 1996). In addition, under the same pH conditions, SACOL0366 a gene encoding for phage terminase small subunit was also up-regulated together with *yoz*M (a prophage-derived-like uncharacterized gene in *Bacillus subtilis*) and several other moderately up-regulated (1.55–1.82 times, see Supplementary Table S1) *S. aureus* phage associated proteins, like a hydrolase and a head protein of phage phi-11, a protein of phage phi-13, a phage anti repressor protein, a conserved phage associated protein, a hypothetical pathogenicity island, and a couple of phage associated proteins similar to those of other bacteria (Supplementary Table S1). Since the small terminase subunit forms a nucleoprotein structure that helpsto position the terminase large subunit at the packaging initiation site that interacts with the double-stranded phage DNA, its over-expression in combination with that of all the above mentioned phage-associated proteins BF cells under acidic conditions is somehow alarming and should be taken into consideration for appropriate use of disinfectants and sanitizers against *S. aureus.* Equally important seems to be the over-expression of *tar*S in planktonic cells at pH5 because TarS is a glycosyl transferase (member of the operon *ispD* > 0241 > 0242 > *tarS*) which glycosylates cell wall teichoic acid polymers, a process that is specifically responsible for methicillin resistance in MRSA (Sobhanifar et al., 2016). The over-expression in the same cells of three genes associated with stress responses (*hch*A, *rec*F and *hup*), is understood under the view of their functions. HchA, a chaperone nucleoid-associated protein H-NS, is known to repress transcription by forming extended DNA-H-NS complexes and capturing early unfolding intermediates under prolonged conditions of severe stress, finally releasing them when cells return to physiological conditions (Mujacic et al., 2004). RecF is a DNA replication and repair protein that can be used by the cell for repairing acidic stress-induced DNA damage, and in combination with the histone-like DNA-binding protein Hup which is capable of wrapping DNA to stabilize it, prevent its denaturation under extreme environmental conditions (Castro et al., 2011). Hup has been also found to repress the *E. coli bgl* operon (Dole et al., 2004), and according to our results a similar association between the two loci seems to exist in *S. aureus*. Under alkaline conditions in planktonic cells, two more cell wall associated genes, *mur*AB and *lyt*M, were down-regulated, along with *par*C, *yoz*E and NP_72897. MurAB, is the UDP-N-acetylglucosamine 1-carboxyvinyltransferase 2, which is involved in glycan synthesis and is known to be over-expressed in *Streptococcus suis* BFs growing under normal conditions (Wang et al., 2012), and LytM - also down-regulated in BF cells at pH5- is the peptidoglycan hydrolase previously considered as the only autolysin of *S. aureus*. Its role as an autolysin has recently been disputed as it was proved to be an early exponential phase protein whose expression was down-regulated by Agr, but still indicates that LytM plays an important role in BF development (Singh et al., 2010). YozE, a hypothetical YozE_SAM_like protein, belongs to a family of proteins with a four-helix motif similar to sterile alpha motif (SAM) domains and is likely to involve binding to DNA (Swapna et al., 2012). NP_72897 is a putative cell division protein, and ParC, is the DNA topoisomerase IV subunit A, responsible for relaxing supercoiled DNA and very important in bacteria replication, where the circular chromosome becomes catenated or linked. In addition, ParC is known to bind to the *ica* cluster, which is involved in the extracellular matrix production (Jefferson et al., 2004). Therefore, it becomes evident that under alkaline stress the planktonic cells are slowing down chromosome replication and cell division in an effort to focus on functions of defense mechanisms against cell damage.

During BF maturation and at the exponential growth phase *S. aureus* normally produces several surface binding proteins, which are subsequently secreted. The surface binding proteins IsdC, MntC, SsaA, SasA, the substrate binding proteins MapW, Efb, Emp, Scc, EpiG, Pls and the envelope and membrane associated proteins TpgX, NP_437451,NP_406103 were all differentially expressed. In BF cells grown at pH9, apart from Vwb and SceD, the cell wall surface anchor protein involved in heme uptake, IsdC, was also up-regulated. IsdC induces BF formation in *S. lugdunensis* grown under iron limitation (Missineo et al., 2014), yet under these conditions *isd*C and a variety of virulence factors are repressed in *S. aureus* (Hammer and Skaar, 2011). Thus, the absence of induction in the expression of any of the known virulence genes under alkaline conditions is most likely due to the simultaneous up-regulation of the global regulator CodY, which is known to negatively regulate virulence gene expression (Majerczyk et al., 2008). The only other cell surface associated protein that was down-regulated in BF cells grown at pH9 was MapW. The same protein was down-regulated under acidic conditions in both BF and planktonic cells. MapW, is a cell surface protein with no LPXTG sequence, which is not recognized and linked to the peptidoglycan by a sortase, but it is released by digestion with lysostaphin. It does not bind to soluble extracellular matrix proteins but functions as an endogenous adhesion substrate in the attachment to plastic surfaces and eukaryotic cells via interaction with staphylococcal surface adhesions (Kreikemeyer et al., 2002). Its down-regulation in both BF cells and planktonic cells strongly suggests that this protein can only be involved in cell attachment to surfaces at neutral pH environments. Together with EbsB, a hypothetical Map-like protein, the fibrinogen-binding protein Efb, and a putative protein (NP_406103) were over-expressed in BF cells at pH5. The hypothetical Map-like protein appears unique as it has no significant similarity with any of the known Map proteins of *S. aureus*. However, as map-like proteins contain up to six MAP domains, each containing a 31-residue sub-domain sharing striking sequence homology with a segment present in the peptide binding groove of the beta chain of the MHC class II proteins from different mammalian species, it is expected to have a different binding ability than MapW. Efb is a secreted virulence factor that helps the pathogen to evade human neutrophils, impairs wound healing and inhibits the formation of platelet-leukocyte complexes (Posner et al., 2016). The NP_406103 putative membrane protein shows some similarity with *gnt*R gene of other *S. aureus* strains, a gene coding for glucokinase. On the contrary, EpiG/BsaG, another extracellular surface associated protein, the epidermin immunity protein F was down-regulated in BF cells. Thus, this differential expression of extracellular surface proteins under the same pH stress is thought to reflect to the influence of these conditions on the ability/inability of cells to adhere on the polystyrene surface. Since EpiG and Scc were found to interact with biotic and abiotic surfaces in a zinc-dependent way (Geoghegan et al., 2013; Nakakido et al., 2014), reducing the availability of zinc ions could lead to the development of novel therapeutic or disinfection strategies for controlling *S. aureus* infections.

In planktonic cells MntC, a conserved manganese-binding surface protein and an ABC (ATP-binding cassette) transporter system component was down-regulated under both pH regimes. Using ELISA tests it was recently demonstrated that MntC is also an ion-scavenging factor with a marked ability to bind to several extracellular matrix and coagulation cascade components, including laminin, collagen type IV, cellular and plasma fibronectin, plasminogen and fibrinogen, hence a potential virulence factor (Salazar et al., 2014). Therefore, its down-regulation at both pH conditions is likely due to iron repletion, as in the case of *S. epidermidis* which was found to withstand higher variations in iron availability when grown planktonically (Oliveira et al., 2017). In planktonic cells grown under alkaline conditions, TpgX, Emp and SsaA were over-expressed. TpgX is a hypothetical protein which following a proteomic profiling of *S. aureus* was shown to be cell envelope-associated (Resch et al., 2006). Emp, a secretory extracellular matrix and plasma binding protein, is an adhesin that displays a broad binding specificity to the host cell extracellular matrix proteins fibronectin, fibrinogen, collagen, and vitronectin (McGavin et al., 1993). Evidently, the up-regulation of these two proteins mediates the adherence on the abiotic surface in spite of the alkaline environment. SsaA, under normal pH conditions is expressed at slightly higher levels in BF cells than in planktonic cells (Resch et al., 2005). Therefore, its up-regulation at pH9 in planktonic cells, should be taken seriously into consideration because as an antigen associated with *S. aureus* surface and staphyloxanthin biosynthesis is considered as a virulence factor. Of equal interest is the up-regulation in planktonic cell growing at pH5 of two genes involved in *S. aureus* virulence, *pls* and *sas*A. As mentioned before, SasA mediates the direct binding of *S*. *aureus* to platelets and contributes to infective endocarditis. The plasmin-sensitive protein gene *pls* which was found to be a virulence factor in mouse septic arthritis model is encoded by the staphylococcal cassette chromosome *mec* type I in MRSA that also encodes the methicillin-conferring *mec*A and further genes and has been found to stimulate BF formation (Bleiziffer et al., 2017). Finally, five more proteins associated with cell surface IsaB, Scc, NP_437451 and chaperones PrsaA, Hfq, were down-regulated in planktonic cells grown under acidic conditions. NP_437451 is a putative membrane transport protein, associated with cell surface. IsaB is an extracellular nucleic acid binding protein with no sequence specificity, which elicits an immune response during septicemia and is generally classified as a virulence factor. However, its role in virulence has not been defined yet (Mackey-Lawrence and Jefferson, 2013). Scc is a fibrinogen-binding protein which facilitates attachment to fibrinogen during colonization of biotic surfaces which is over-expressed during BF formation *in vitro* and is crucial for the colonization of medical devices by healthcare *S. aureus* strains (Geoghegan et al., 2013). PrsA is a post-translocational chaperone lipoprotein that is involved in both glycopeptide and oxacillin resistance in *S. aureus*. More specifically, disruption of *prs*A leads to notable alterations in the sensitivity to glycopeptides and dramatically decreases the resistance of *S. aureus* COL (MRSA) to oxacillin (Jousselin et al., 2012). Hfq, an RNA chaperone that binds small regulatory RNAs and mRNAs, negatively regulates translation in response to envelope stress, environmental stress and changes in metabolite concentrations and upon over-expression decreases persister cell formation (Guisbert et al., 2007). The down-regulation of all these genes involved in virulence and antibiotic resistance in planktonic cells, is rather promising because it confirms that alkaline conditions can be safely used as disinfectants to prevent BF formation on food processing and/or indwelling medical devices. Finally, the down-regulation of the putative monoamine oxidase regulator gene *mao*C1, at both pH9 and pH5, is rather interesting in view of its putative function. Bacterial cells respond to monoamine compounds, such as tyramine, dopamine, octopamine, or norepinephrine, and induce the syntheses of tyramine oxidase encoded by *tyn*A and *mao*A. The monoamine oxidase regulator gene *moa*R of several bacteria was found to play a central role in the positive regulation of the expression of the monoamine regulon (*moa*) including the *ats*BA, *mao*CA, *moa*EF and *tyn* operons (Murooka et al., 1996). Thus, a similar role may be envisaged for the *S. aureus mao*C1 and its importance may be associated with the enzymatic activity of FabI in the essential fatty acid biosynthesis pathway, as studies with antisense RNA have shown (Ji et al., 2004).

Staphylococcal enterotoxins are important causative agents in staphylococcal toxic shock syndrome and food poisoning (Orwin et al., 2001). Staphylococcal superantigen-like (SSL) proteins are encoded by a cluster of fourteen *ssl* genes and contribute to the *S. aureus* virulence. Despite their structural similarity to superantigens, SSLs do not bind to T-cell receptors or major histocompatibility complex class II molecules but they target components of innate immunity and myeloid cells (Hermans et al., 2012). In this context, it is important to underline that no exotoxin or enterotoxin genes were over-expressed under highly alkaline or acidic conditions in both BF and planktonic cells (Tables 3, 4). This is in sharp contrast with results from other researchers working with cells growing at pH7 who have found genes that can lead to toxic shock and sepsis as the exotoxins 6 (*ssl*1) and 15 (*ssl*11) over-expressed in both BF and planktonic cells, and in addition, in planktonic cells the genes encoding exotoxin 3(*ssl*14) and enterotoxin K (*sek)* also up-regulated (Aguilar et al., 2017). This indicates that the pathogen is not launching a damaging offensive against host tissue when defending against a highly alkaline or acidic environment, a finding that could have direct clinical importance in the case of vaginal *S. aureus* infections, since the pH of this organ is low (between 3.8 and 4.5) due to its acidic secretions. Also, *S. aureus* is known to invade and survive for a short amount of time in the lysosomal compartment of non-phagocytic cells (pH 4.5 to 5.5), before escaping into the cytosol (Anderson et al., 2010). Although with a hypothetical function, the up-regulation of *mdl*B in planktonic cells at both pHs, merits particular attention as it codes for a putative ABC transporter permease and ATP–binding protein which may be exporting toxin(s).

As for genes involved directly or indirectly in antibiotic resistance of MRSAs we recorded four genes that were differentially expressed only in planktonic cells. Up-regulated were the genes *fem*A, at both pHs, *sep*A at pH9, *pls* at pH5, and down-regulated was *mde*A at pH9. *Fem*A, codes fo FEMA, an aminoacyl-transferase which catalyzes the formation of the pentaglycine interpeptide bridge in *S. aureus* peptidoglycan and is considered as a factor influencing the level of methicillin resistance. It also strengthens the cell wall and is involved in dormancy (Savijoki et al., 2016). When *femA* was inactivated, mutant cells had a reduced peptidoglycan glycine content, reduced cell wall turnover, reduced whole-cell autolysis, and increased sensitivity toward β-lactam antibiotics (Maidhof et al., 1991). This is in agreement with the up-regulation of the capsule biosynthesis and autolytic enzymes observed in our study. Interestingly, acidic pH was found to restore susceptibility of methicillin-resistant *Staphylococcus aureus* to β-lactam antibiotics (Lemaire et al., 2008). Gene *sepA* encodes a drug efflux protein with four predicted transmembrane segments, which proved to be a multi-drug resistance gene when cloned from *S. aureus* to *E. coli* conferring the reduction of susceptibility to acriflavine and the acceleration of ethidium bromide efflux from the *E. coli* cells (Narui et al., 2002). As mentioned before, Pls is a methicillin-resistant surface protein precursor which could be involved in methicillin resistance by *S. aureus* (Bleiziffer et al., 2017), and a similar role may be attributed to the down-regulated at pH5 hypothetical *mdeA/emrB* gene which codes for a multi-drug resistance-related transporter.

## Conclusion

Our results show that when *S. aureus* COL grows under highly acidic or alkaline conditions it attempts to respond to the resulting stress, repair its cell wall, protect itself by forming BF when an appropriate surface is provided, strengthen the BF by release of extracellular DNA and boost its resistance to antibiotics. In the meantime, it reduces its virulence (e.g., toxin production), as it has entered a defensive mode.

Interestingly, although the exact role in *S. aureus* BF formation of many of the transcription factors, stress response systems and adhesion proteins that were described above are not fully demonstrated yet, their involvement in BF formation is rather apparent. By examining the effect of alkaline and acidic pH on the gene expression of MRSA BF cells for first time, we have contributed important data to the understanding of cellular adjustments that might influence colonization, virulence and antibiotic resistance in this defensive growth mode. Overall, our results showed that *S. aureus* COL can easily grow at highly alkaline and acidic environments and led to the identification of several genes that were differentially expressed under these conditions and could be involved in stress response, virulence and antibiotic resistance pathways in this important pathogen. Understanding how the pathogen survives and responds under these conditions will certainly influence the design of better treatment or disinfection strategies in the future.

## Data Availability Statement

The datasets generated and analyzed for this study can be found in the GEO repository (GSE138075) at: https://www.ncbi.nlm.nih.gov/geo/query/acc.cgi?acc=GSE138075.

## Author Contributions

GE, MT, and KP conceived and designed the experiments. GE performed the experiments. GE, GT, MT, and KP analyzed the data. GE, MT, and KP wrote the manuscript.

## Conflict of Interest

The authors declare that the research was conducted in the absence of any commercial or financial relationships that could be construed as a potential conflict of interest.

## Footnotes

1 https://www.thermofisher.com/order/catalog/product/900514?SID=srch-srp-900514

## Supplementary Material

TABLE S1 Diffentially-expressed genes at different conditions (alkaline or acidic pH; biofilm or planktonic growth), with a log^2^ ratio between 1.5 and 2 or –1.5 and –2.
