# Supplementary figures and images for: Transcriptomic Adjustments of Staphylococcus aureus COL (MRSA) Forming Biofilms Under Acidic and Alkaline Conditions
Source: Front Microbiol. 2019 Oct 18;10:2393. doi: 10.3389/fmicb.2019.02393 (PMC6813237; doi:10.3389/fmicb.2019.02393)

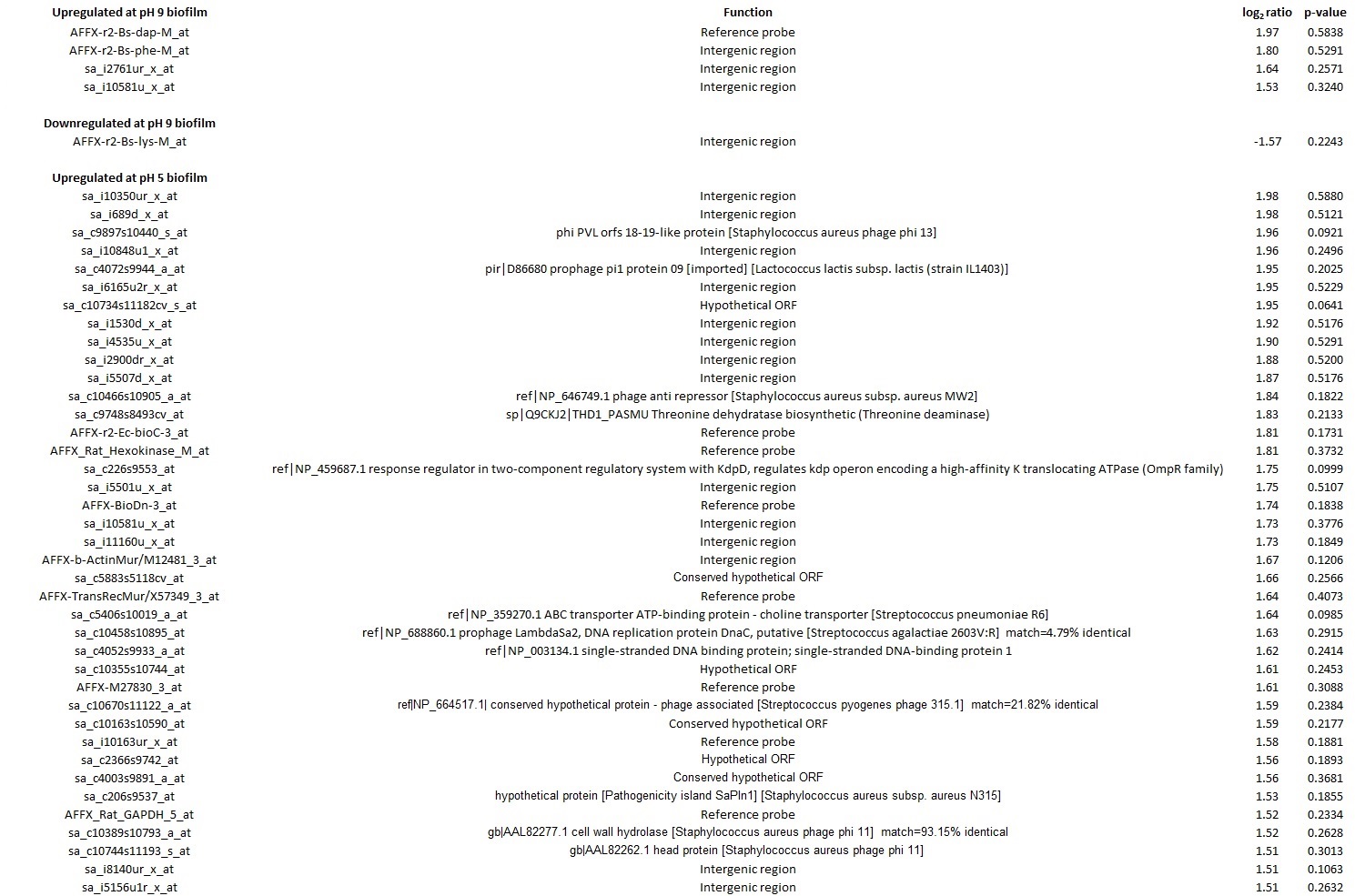

Supplement: Supplementary file 1 [file Image_1.JPEG]

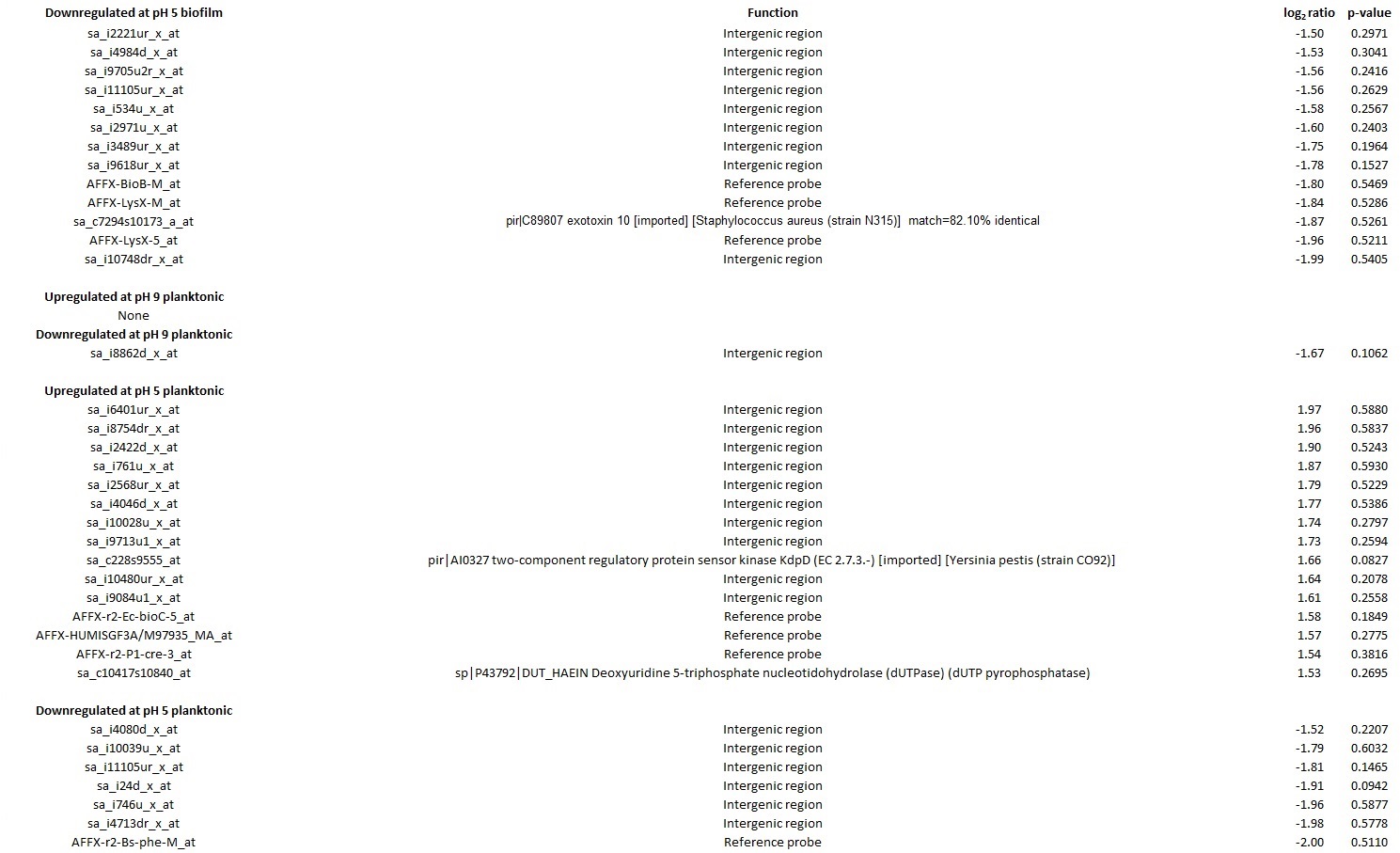

Supplement: TABLE S1 — Diffentially-expressed genes at different conditions (alkaline or acidic pH; biofilm or planktonic growth), with a log2 ratio between 1.5 and 2 or –1.5 and –2. [file Image_2.JPEG]
